# Supplementary figures and images for: Molecular time estimates for the Lagomorpha diversification
Source: PLoS One. 2024 Sep 6;19(9):e0307380. doi: 10.1371/journal.pone.0307380 (PMC11379240; doi:10.1371/journal.pone.0307380)

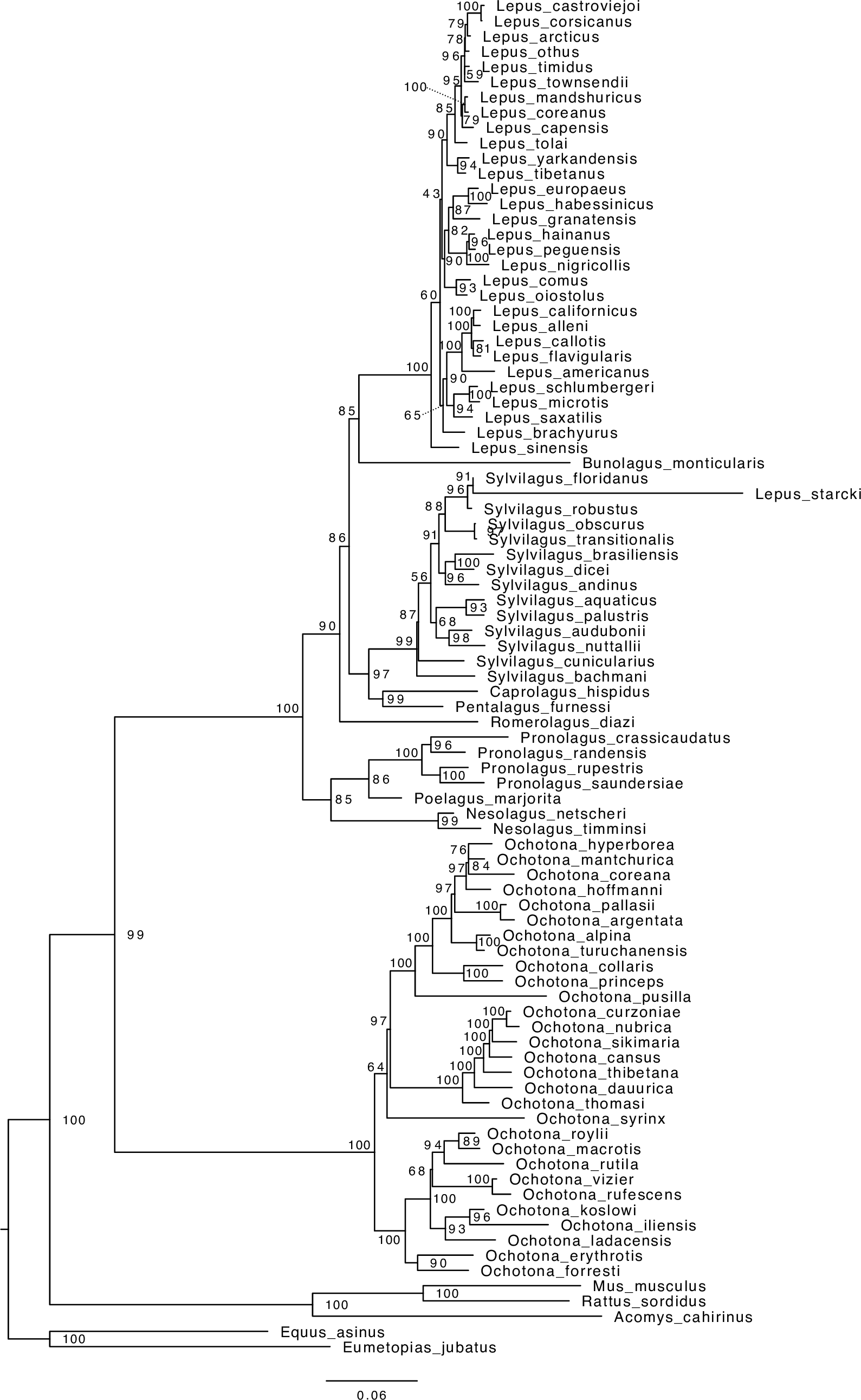

Supplement: S1 Fig — Maximum likelihood tree generated using 79 terminals and 5 mitochondrial genes. Nodes with bootstrap values lower than 100 shown. In this topology, all species (including those that were considered rogue taxa) were kept. (TIF) [file pone.0307380.s001.tif]
