# Supplementary material for: Molecular time estimates for the Lagomorpha diversification
Source: PLoS One. 2024 Sep 6;19(9):e0307380. doi: 10.1371/journal.pone.0307380 (PMC11379240; doi:10.1371/journal.pone.0307380)
Supplement: S1 Table — (DOCX) [file pone.0307380.s002.docx]

| **Family** | **Subgenus** | **Species** |
| --- | --- | --- |
| Leporidae |  | *Bunolagus monticularis* |
| Leporidae |  | *Caprolagus hispidus* |
| Leporidae |  | *Lepus alleni* |
| Leporidae |  | *Lepus americanus* |
| Leporidae |  | *Lepus arcticus* |
| Leporidae |  | *Lepus brachyurus* |
| Leporidae |  | *Lepus californicus* |
| Leporidae |  | *Lepus callotis* |
| Leporidae |  | *Lepus capensis* |
| Leporidae |  | *Lepus castroviejoi* |
| Leporidae |  | *Lepus comus* |
| Leporidae |  | *Lepus coreanus* |
| Leporidae |  | *Lepus corsicanus* |
| Leporidae |  | *Lepus europaeus* |
| Leporidae |  | *Lepus flavigularis* |
| Leporidae |  | *Lepus granatensis* |
| Leporidae |  | *Lepus hainanus* |
| Leporidae |  | *Lepus mandshuricus* |
| Leporidae |  | *Lepus microtis* |
| Leporidae |  | *Lepus oiostolus* |
| Leporidae |  | *Lepus othus* |
| Leporidae |  | *Lepus peguensis* |
| Leporidae |  | *Lepus saxatilis* |
| Leporidae |  | *Lepus sinensis* |
| Leporidae |  | *Lepus timidus* |
| Leporidae |  | *Lepus tolai* |
| Leporidae |  | *Lepus townsendii* |
| Leporidae |  | *Nesolagus netscheri* |
| Leporidae |  | *Nesolagus timminsi* |
| Ochotonidae | *Pika* | *Ochotona alpina* |
| Ochotonidae | *Pika* | *Ochotona argentata* |
| Ochotonidae | *Ochotona* | *Ochotona cansus* |
| Ochotonidae | *Pika* | *Ochotona collaris* |
| Ochotonidae | *Pika* | *Ochotona coreana* |
| Ochotonidae | *Ochotona* | *Ochotona curzoniae* |
| Ochotonidae | *Ochotona* | *Ochotona dauurica* |
| Ochotonidae | *Conothoa* | *Ochotona erythrotis* |
| Ochotonidae | *Conothoa* | *Ochotona forresti* |
| Ochotonidae | *Pika* | *Ochotona hoffmanni* |
| Ochotonidae | *Pika* | *Ochotona hyperborea* |
| Ochotonidae | *Conothoa* | *Ochotona iliensis* |
| Ochotonidae | *Conothoa* | *Ochotona koslowi* |
| Ochotonidae | *Conothoa* | *Ochotona ladacensis* |
| Ochotonidae | *Conothoa* | *Ochotona macrotis* |
| Ochotonidae | *Pika* | *Ochotona mantchurica* |
| Ochotonidae | *Ochotona* | *Ochotona nubrica* |
| Ochotonidae | *Pika* | *Ochotona pallasi* |
| Ochotonidae | *Pika* | *Ochotona princeps* |
|  | *Lagotona* | *Ochotona pusilla* |
| Ochotonidae | *Conothoa* | *Ochotona roylii* |
| Ochotonidae | *Conothoa* | *Ochotona rufensis* |
| Ochotonidae | *Conothoa* | *Ochotona rutila* |
| Ochotonidae | *Ochotona* | *Ochotona sikimaria* |
| Ochotonidae | *Alienauroa* | *Ochotona syrinx* |
| Ochotonidae | *Ochotona* | *Ochotona thibetana* |
| Ochotonidae | *Ochotona* | *Ochotona thomasi* |
| Ochotonidae | *Pika* | *Ochotona turuchanensis* |
| Ochotonidae | *Conothoa* | *Ochotona vizier* |
| Leporidae |  | *Oryctolagus cuniculus* |
| Leporidae |  | *Pentalagus furnessi* |
| Leporidae |  | *Pronolagus crassicaudatus* |
| Leporidae |  | *Pronolagus randensis* |
| Leporidae |  | *Pronolagus rupestris* |
| Leporidae |  | *Pronolagus saundersiae* |
| Leporidae |  | *Romerolagus diazi* |
| Leporidae |  | *Sylvilagus aquaticus* |
| Leporidae |  | *Sylvilagus audubonii* |
| Leporidae |  | *Sylvilagus bachmani* |
| Leporidae |  | *Sylvilagus brasiliensis* |
| Leporidae |  | *Sylvilagus dicei* |
| Leporidae |  | *Sylvilagus floridanus* |
| Leporidae |  | *Sylvilagus nuttallii* |
| Leporidae |  | *Sylvilagus obscurus* |
| Leporidae |  | *Sylvilagus palustris* |
| Leporidae |  | *Sylvilagus robustus* |
| Leporidae |  | *Sylvilagus transitionalis* |
