# Supplementary material for: Molecular time estimates for the Lagomorpha diversification
Source: PLoS One. 2024 Sep 6;19(9):e0307380. doi: 10.1371/journal.pone.0307380 (PMC11379240; doi:10.1371/journal.pone.0307380)
Supplement: S2 Table — (PDF) [file pone.0307380.s003.pdf]

Taxa	SRY	RAG1	ND2	RAG2	PHKA2	DARC	OXA1L	12S	MC1R	IL1RAPL1	TG	TF	TSHB	ND4	PRKC1	SPTBN	MGF	CYTB	COX1
Acomys_cahirinus	-	-	-	-	-	-	-	NC_020758	-	-	-	-	-	NC_020758	-	-	AY292745.1	NC_020758	NC_020758
Caprolagus_hispidus	-	-	-	-	-	-	-	AY292693.1	-	-	AY292838.1	-	-	-	AY292770.1	AY292812.1	AY292743.1	AY292719.1	-
Equus_asinus	-	-	-	-	-	-	-	OQ868402.1	-	-	-	-	-	OQ868402.1	-	-	-	OL660218.1	OQ868402.1
Eumetopias_jubatus	-	-	-	-	-	-	-	GU475464.1	-	-	-	-	-	GU475464.1	-	-	-	AB362485.1	GU475464.1
Lepus_alleni	-	-	-	-	-	-	-	-	-	-	-	-	-	-	-	-	-	AF010156.1	KT308120.1
Lepus_americanus	KM261382.1	-	-	-	-	JN036931.1	JN037149.1	-	-	-	AY292852.1	JN037069.1	AY292681.1	-	AY292784.1	AY292826.1	KM261027.1	HQ596459.1	KT308121.1
Lepus_arcticus	-	-	-	-	-	JN036927.1	JN037144.1	-	-	-	-	JN037064.1	JN037117.1	-	JN037011.1	JN037038.1	JN036982.1	AF010153.1	JF443245.1
Lepus_brachyurus	LC132589.1	-	-	-	LC132528.1	-	-	-	AB824136.1	-	LC132043.1	-	AB595499.1	-	-	AB595769.1	LC131946.1	AB158509.1	LC619116.1
Lepus_californicus	KM261406.1	-	MG137399.1	-	-	KM260987.1	JN037154.1	-	LR989848.1	-	AY292850.1	JN037072.1	AY292679.1	-	AY292782.1	AY292824.1	KM261073.1	AY292731.1	GU670578.1
Lepus_callotis	-	-	MG137394.1	-	-	-	-	-	-	-	-	-	-	-	-	-	-	AF010158.1	-
Lepus_capensis	-	-	KX574630.1	-	-	MK776117.1	JN037141.1	AY292706.1	HM452335.1	-	AY292851.1	AY176277.1	AY292680.1	HM232895.1	AY292783.1	AY292825.1	AY292756.1	AF157457.1	KF153030.1
Lepus_castroviejoi	-	-	MG137348.1	-	-	FJ811759.1	FJ811604.1	-	HQ005374.1	-	-	AY176268.1	JN037096.1	-	FJ811679.1	FJ811652.1	-	AY176235.1	-
Lepus_comus	-	-	-	-	-	-	-	AY745179.1	-	-	HM233354.1	HM233536.1	-	HM232950.1	HM233518.1	HM233500.1	HM233614.1	AJ241613.1	HM233109.1
Lepus_coreanus	LC132579.1	-	-	-	LC132503.1	-	-	-	LC132309.1	-	LC132019.1	-	LC132115.1	-	-	LC132214.1	LC131922.1	AB687531.1	KP993120.1
Lepus_corsicanus	-	-	MG137349.1	-	-	JN036919.1	JN037137.1	-	HM452333.1	-	-	AY176270.1	JN037103.1	-	-	-	-	AF157463.1	-
Lepus_europaeus	FJ811533.1	-	GU085216.1	-	KF564346.1	FJ811758.1	FJ811596.1	KF781316.1	HM452330.1	-	MH115262.1	AY176259.1	JN037088.1	KU250117.1	FJ811671.1	FJ811645.1	FJ811698.1	AF010161.1	EU623451.1
Lepus_flavigularis	-	-	MG137403.1	-	-	-	-	-	-	-	-	-	-	-	-	-	-	HQ596475.1	KT308122.1
Lepus_granatensis	-	-	MG137380.1	-	JF299165.1	FJ811747.1	FJ811588.1	-	HM452337.1	-	MK776415.1	AY176251.1	JN037080.1	-	FJ811666.1	FJ811640.1	JN036970.1	AF157465.1	-
Lepus_habessinicus	-	-	MG137350.1	-	-	-	-	-	-	-	-	-	-	-	-	-	-	AF157455.1	MN326050.1
Lepus_hainanus	-	-	-	-	-	-	-	-	-	-	HM233360.1	HM233552.1	-	HG763836.1	HM233515.1	HM233486.1	HG763851.1	AY599077.1	HG763835.1
Lepus_mandshuricus	LC132585.1	-	-	-	LC132499.1	-	-	-	LC132305.1	-	HM233388.1	HM233545.1	LC132111.1	HM232876.1	HM233522.1	HM233498.1	LC131917.1	AY650894.1	HM233118.1
Lepus_microtis	-	-	-	-	-	-	-	KJ192554.1	-	-	-	-	-	-	-	-	-	KJ193381.1	KJ192836.1
Lepus_nigricollis	-	-	-	-	-	-	-	-	-	-	-	-	-	-	-	-	-	MT039015.1	-
Lepus_oiostolus	-	-	-	-	-	-	-	AY745178.1	-	-	HM233370.1	HM233551.1	-	HM232944.1	HM233510.1	HM233504.1	HM233625.1	AJ279426.1	HM233103.1
Lepus_othus	-	-	-	-	-	JN036930.1	JN037147.1	MN175786.1	-	-	-	JN037068.1	JN037119.1	-	JN037014.1	JN037041.1	JN036986.1	AF010154.1	-
Lepus_peguensis	-	-	-	-	-	-	-	-	-	-	-	-	-	HG763837.1	-	-	HG763852.1	HG763842.1	HG763831.1
Lepus_saxatilis	-	-	MG137404.1	-	-	-	-	-	HQ005376.1	-	AY292849.1	AY176279.1	AY292678.1	-	AY292781.1	AY292823.1	-	AF009731.1	-
Lepus_schlumbergeri	-	-	-	-	-	-	-	MN175781.1	-	-	-	MN175904.1	-	-	MN175884.1	MN175918.1	MN175764.1	MN175757.1	MN175849.1
Lepus_sinensis	-	-	-	-	-	-	-	OM334908.1	-	-	HM233367.1	HM233538.1	-	HM232966.1	HM233514.1	HM233489.1	HM233565.1	AJ279418.1	HM233195.1
Lepus_tibetanus	-	-	-	-	-	-	-	-	-	-	-	MN098961.1	-	OL416132.1	-	-	-	MH170198.1	OL374006.1
Lepus_timidus	LC132568.1	-	MG137410.1	-	JF299177.1	FJ811765.1	FJ811608.1	OQ508154.1	HM452331.1	-	AY292847.1	AY176271.1	AY292676.1	HM232871.1	AY292779.1	AY292821.1	FJ811717.1	AB687499.1	KX859258.1
Lepus_tolai	-	-	-	-	-	-	-	-	-	-	-	-	-	OP302843.1	-	-	-	ON470370.1	OP594716.1
Lepus_townsendii	KM261426.1	-	-	-	-	JN036937.1	JN037155.1	-	LR989847.1	-	AY292848.1	JN037075.1	AY292677.1	-	AY292780.1	AY292822.1	KM261104.1	AF009733.1	-
Lepus_yarkandensis	-	-	-	-	-	-	-	-	-	-	HM233366.1	HM233543.1	-	HM232929.1	HM233517.1	HM233488.1	HM233566.1	AY675173.1	KF153031.1
Mus_musculus	-	-	-	-	-	-	-	EU450583.1	-	-	-	-	-	EU450583.1	-	-	-	KX790793.1	EU450583.1
Nesolagus_netscheri	-	-	-	-	-	-	-	AY292709.1	-	-	AY292854.1	-	AY292683.1	-	AY292786.1	-	AY292759.1	-	-
Nesolagus_timminsi	-	-	-	FM162112.1	-	-	-	AY292710.1	-	-	AY292855.1	-	AY292684.1	-	AY292787.1	AY292828.1	AY292760.1	MT986855.1	-
Ochotona_alpina	-	KY025147.1	-	KY025241.1	-	KP293021.1	KP293083.1	-	LR990398.1	KP293062.1	-	-	KP293165.1	AF273130.1	KP293123.1	KP293144.1	KT317762.1	AF273009.1	DQ347413.1
Ochotona_argentata	-	-	-	-	-	-	-	-	-	-	-	-	-	-	-	-	KT317769.1	-	KT317752.1
Ochotona_cansus	-	KT779435.1	-	KY025242.1	-	-	KY025073.1	-	-	KY024981.1	-	-	-	AF273125.1	MH361455.1	-	MH361408.1	AF273003.1	KC709688.1
Ochotona_collaris	-	-	-	-	-	-	-	-	-	-	-	-	-	EU549747.1	-	-	-	AF176578.1	KR732589.1
Ochotona_coreana	-	-	-	-	-	-	-	-	-	-	-	-	-	-	-	-	-	EF567060.1	-
Ochotona_curzoniae	-	KT779437.1	-	KY025245.1	-	-	KY025077.1	KF038167.1	-	KY024984.1	-	-	OP250896.1	AF273122.1	KT317750.1	OP250870.1	MH361407.1	AF176581.1	JX962316.1
Ochotona_dauurica	-	OM792900.1	-	OM792932.1	-	-	-	-	LR990392.1	MH361549.1	-	-	KP293168.1	AF273134.1	MH361445.1	-	MH361398.1	AF273000.1	DQ347426.1
Ochotona_erythrotis	-	OM792879.1	-	OM792912.1	-	-	OM792855.1	-	-	OM792800.1	-	-	-	AF273121.1	-	-	OM792822.1	AF272999.1	OM832781.1
Ochotona_forresti	-	KY025165.1	-	KY025259.1	-	-	KY025088.1	-	-	KY024999.1	-	-	-	-	-	-	OM792811.1	KX989537.1	OM832770.1
Ochotona_hoffmanni	-	-	-	-	-	KP293025.1	KP293086.1	-	-	KP293066.1	-	-	KP293169.1	-	KP293127.1	KP293149.1	KT317783.1	HM594687.1	-
Ochotona_hyperborea	-	KY025180.1	-	KY025274.1	-	-	KY025095.1	AY012127.1	LR990397.1	KY025013.1	-	-	OP250895.1	AF273115.1	OP250850.1	OP250869.1	KT317761.1	AF176582.1	DQ347443.1
Ochotona_iliensis	-	-	-	-	-	-	-	-	-	-	-	-	-	-	-	-	-	AY191824.1	-
Ochotona_koslowi	-	-	-	-	-	-	-	-	-	-	-	-	-	AF273116.1	-	-	-	AF272993.1	-
Ochotona_ladacensis	-	OM792894.1	-	OM792927.1	-	-	OM792863.1	-	-	OM792809.1	-	-	-	AF273114.1	-	-	OM792839.1	AF272992.1	-
Ochotona_macrotis	-	OM792872.1	-	OM792905.1	-	-	OM792849.1	-	LR990396.1	OM792794.1	-	-	-	-	-	-	OM792815.1	MN076081.1	OM832777.1
Ochotona_mantchurica	-	-	-	-	-	KP293028.1	KP293090.1	-	-	KP293070.1	-	-	KP293173.1	-	KP293131.1	KP293152.1	KT317785.1	DQ335518.1	DQ347449.1
Ochotona_nubrica	-	-	-	-	-	-	-	-	-	MH361556.1	-	-	-	AF273113.1	MH361447.1	-	MH361404.1	AF272991.1	MH361498.1
Ochotona_pallasii	-	-	-	-	-	KP293030.1	KP293092.1	-	-	KP293072.1	-	-	KP293175.1	AF273118.1	KP293133.1	KP293154.1	KT317770.1	AF272990.1	DQ347452.1
Ochotona_princeps	-	JQ073183.1	-	KY025281.1	-	KP293032.1	KY025101.1	-	-	KP293074.1	AY292834.1	-	AY292664.1	AF273112.1	AY292766.1	AY292809.1	EU591095.1	AF176579.1	KR732591.1
Ochotona_pusilla	-	OM792896.1	-	OM792929.1	-	KP293033.1	KP293095.1	-	LR990393.1	KP293075.1	-	-	KP293179.1	-	KP293136.1	KP293158.1	KT317760.1	HM366945.1	DQ347457.1
Ochotona_roylii	-	KY025192.1	-	OM792920.1	-	-	OM792861.1	-	-	KY025023.1	-	-	-	GU825953.1	-	-	OM792832.1	AF272988.1	KY024836.1
Ochotona_rufescens	-	KY025193.1	-	KY025284.1	-	-	KY025105.1	-	-	KY025024.1	-	-	OP250877.1	-	-	OP250859.1	OM792834.1	KF835429.1	OM832787.1

Taxa	SRY	RAG1	ND2	RAG2	PHKA2	DARC	OXA1L	12S	MC1R	IL1RAPL1	TG	TF	TSHB	ND4	PRKC1	SPTBN	MGF	CYTB	COX1
Ochotona_rutila	-	OM792880.1	-	OM792913.1	-	KP293037.1	KP293098.1	-	-	KP293078.1	-	-	KP293182.1	-	-	KP293161.1	MH361391.1	AF515733.1	MH361537.1
Ochotona_sikimaria	-	KT779441.1	-	KT779450.1	-	-	-	-	-	-	-	-	-	KF548186.1	-	-	-	KT779470.1	-
Ochotona_syrinx	-	KY025229.1	-	KY025320.1	-	-	KY025137.1	-	-	KY025060.1	-	-	-	-	-	-	MH361392.1	KY024968.1	MH361501.1
Ochotona_thibetana	-	KY025235.1	-	KY025326.1	-	-	KY025145.1	-	-	KY025066.1	-	-	-	AF273110.1	-	-	MH361424.1	AF176580.1	MH361509.1
Ochotona_thomasi	-	-	-	-	-	-	-	-	-	MH361581.1	-	-	-	AF273111.1	-	-	MH361421.1	AF272987.1	MH361503.1
Ochotona_turuchanensis	-	-	-	-	-	KP293039.1	KP293100.1	-	-	KP293080.1	-	-	KP293184.1	-	KP293142.1	KP293163.1	KT317765.1	DQ335488.1	DQ347461.1
Ochotona_vizier	-	-	-	-	-	-	-	-	-	OP250808.1	-	-	OP250874.1	-	-	OP250856.1	-	OP227090.1	-
Pentalagus_furnessi	-	-	-	-	-	-	-	AB058603.1	LR990389.1	-	AY292839.1	-	AY292668.1	-	AY292771.1	AY292813.1	AY292744.1	LC107934.1	-
Poelagus_marjorita	-	-	-	-	-	-	-	-	-	-	AY292835.1	-	-	-	AY292767.1	-	-	-	-
Pronolagus_crassicaudatus	-	-	-	-	-	-	-	AY292714.1	-	-	AY292859.1	-	AY292688.1	-	AY292791.1	AY292832.1	AY292764.1	AY292738.1	-
Pronolagus_randensis	-	-	-	-	-	-	-	AY292713.1	-	-	AY292858.1	-	AY292687.1	-	AY292790.1	AY292831.1	AY292763.1	AY292737.1	-
Pronolagus_rupestris	-	-	-	-	-	-	-	AY292711.1	-	-	AY292856.1	-	AY292685.1	-	AY292788.1	AY292829.1	AY292761.1	AY292735.1	-
Pronolagus_saundersiae	-	-	-	-	-	-	-	AY292712.1	-	-	AY292857.1	-	AY292686.1	-	AY292789.1	AY292830.1	AY292762.1	AY292736.1	-
Rattus_sordidus	-	-	-	-	-	-	-	NC_014871.1	-	-	-	-	-	NC_014871.1	-	-	-	EF186480.1	NC_014871.1
Romerolagus_diazi	-	-	-	-	-	-	-	AB053205.1	LR990394.1	-	AY292853.1	-	AY292682.1	-	AY292785.1	AY292827.1	AY292758.1	MN062112.1	-
Sylvilagus_aquaticus	-	-	-	-	-	-	-	-	-	-	AY292845.1	-	AY292674.1	-	AY292777.1	AY292819.1	-	AY292726.1	-
Sylvilagus_audubonii	-	-	-	-	-	-	-	KU057212.1	-	-	AY292841.1	-	AY292670.1	-	AY292773.1	AY292815.1	-	AY292722.1	GU681786.1
Sylvilagus_brasiliensis	-	-	-	-	-	-	-	KU057219.1	-	-	MH115240.1	-	MH115252.1	-	MH115223.1	MH115230.1	MH115213.1	MH115201.1	JF444945.1
Sylvilagus_dicei	-	-	-	-	-	-	-	KU057251.1	-	-	-	-	-	-	-	-	-	-	-
Sylvilagus_floridanus	-	AY011895.1	-	AY011952.1	-	JN036941.1	JN037159.1	AY012126.1	LR990391.1	-	AY292843.1	AY176280.1	AY292672.1	-	AY292775.1	AY292817.1	-	AF034257.1	JF443513.1
Sylvilagus_nuttallii	-	-	-	-	-	-	-	KU057255.1	LR990395.1	-	AY292842.1	-	AY292671.1	-	AY292774.1	AY292816.1	-	AY292723.1	-
Sylvilagus_obscurus	-	-	-	-	-	-	-	KU057247.1	-	-	AY292844.1	-	AY292673.1	-	AY292776.1	AY292818.1	-	AY292725.1	-
Sylvilagus_palustris	-	-	-	-	-	-	-	KU057249.1	-	-	AY292846.1	-	AY292675.1	-	AY292778.1	AY292820.1	-	AY292727.1	-
Sylvilagus_robustus	-	-	-	-	-	-	-	-	-	-	-	-	OM363047.1	-	OK275709.1	-	-	HQ143449.1	-
Sylvilagus_transitionalis	-	-	-	-	-	-	-	KU057250.1	-	-	-	-	-	-	-	-	-	AF034256.1	-
